# The combination of atezolizumab and BCG in high-risk non-muscle invasive bladder cancer: results of the phase Ib/II BladderGATE clinical trial

**DOI:** 10.1093/oncolo/oyag177

**Published:** 2026-05-08

**Authors:** Félix Guerrero-Ramos, Carmen Gómez-Cañizo, Marta Rodríguez-Izquierdo, Mario Hernández-Arroyo, Alfredo Rodríguez-Antolín, Guillermo de Velasco, Santiago Ponce-Aix, Jesús M Paramio, Cristian Suárez-Cabrera, Carolina Rubio, Lucía Morales, Víctor García-Martínez, Marta Dueñas, Daniel Castellano

**Affiliations:** Department of Urology, Hospital Universitario 12 de Octubre, Avda. Córdoba S/N, Madrid, 28041, Spain; Institute of Biomedical Research, Hospital Universitario 12 de Octubre, Madrid, 28041, Spain; Department of Urology, Hospital Universitario 12 de Octubre, Avda. Córdoba S/N, Madrid, 28041, Spain; Department of Urology, Hospital Universitario 12 de Octubre, Avda. Córdoba S/N, Madrid, 28041, Spain; Department of Urology, Hospital Universitario 12 de Octubre, Avda. Córdoba S/N, Madrid, 28041, Spain; Department of Urology, Hospital Universitario 12 de Octubre, Avda. Córdoba S/N, Madrid, 28041, Spain; Institute of Biomedical Research, Hospital Universitario 12 de Octubre, Madrid, 28041, Spain; Institute of Biomedical Research, Hospital Universitario 12 de Octubre, Madrid, 28041, Spain; Department of Medical Oncology, Hospital Universitario 12 de Octubre, Madrid, 28041, Spain; Department of Medical Oncology, Hospital Universitario 12 de Octubre, Madrid, 28041, Spain; Scientific Coordinator—Oncosur Foundation, Madrid, Spain; Institute of Biomedical Research, Hospital Universitario 12 de Octubre, Madrid, 28041, Spain; Molecular and Translational Oncology Division, Biomedical Innovation Unit, Center for Energy, Environmental and Technological Research (CIEMAT), Madrid, Spain; Biomedical Research Center in Cancer Network (CIBERONC), Madrid, Spain; Molecular and Translational Oncology Division, Biomedical Innovation Unit, Center for Energy, Environmental and Technological Research (CIEMAT), Madrid, Spain; Biomedical Research Center in Cancer Network (CIBERONC), Madrid, Spain; Cellular and Molecular Oncology and Genitourinary Tumor Group. Institute of Biomedical Research, Hospital Universitario, 12 de Octubre, Madrid, Spain; Molecular and Translational Oncology Division, Biomedical Innovation Unit, Center for Energy, Environmental and Technological Research (CIEMAT), Madrid, Spain; Cellular and Molecular Oncology and Genitourinary Tumor Group. Institute of Biomedical Research, Hospital Universitario, 12 de Octubre, Madrid, Spain; Molecular and Translational Oncology Division, Biomedical Innovation Unit, Center for Energy, Environmental and Technological Research (CIEMAT), Madrid, Spain; Institute of Biomedical Research, Hospital Universitario 12 de Octubre, Madrid, 28041, Spain; Molecular and Translational Oncology Division, Biomedical Innovation Unit, Center for Energy, Environmental and Technological Research (CIEMAT), Madrid, Spain; Biomedical Research Center in Cancer Network (CIBERONC), Madrid, Spain; Molecular and Translational Oncology Division, Biomedical Innovation Unit, Center for Energy, Environmental and Technological Research (CIEMAT), Madrid, Spain; Biomedical Research Center in Cancer Network (CIBERONC), Madrid, Spain; Cellular and Molecular Oncology and Genitourinary Tumor Group. Institute of Biomedical Research, Hospital Universitario, 12 de Octubre, Madrid, Spain; Institute of Biomedical Research, Hospital Universitario 12 de Octubre, Madrid, 28041, Spain; Department of Medical Oncology, Hospital Universitario 12 de Octubre, Madrid, 28041, Spain; Biomedical Research Center in Cancer Network (CIBERONC), Madrid, Spain

**Keywords:** atezolizumab, Bacillus Calmette-Guérin, BCG-naïve, clinical trial, non–muscle invasive bladder cancer, recurrence-free survival

## Abstract

**Background:**

BladderGATE evaluated the safety, tolerability, and efficacy of intravenous (IV) atezolizumab combined with intravesical Bacillus Calmette-Guérin (BCG) in patients with BCG-naïve high-risk non-muscle invasive bladder cancer (NMIBC).

**Materials and methods:**

This was a phase Ib/II open-label, nonrandomized, dose de-escalation clinical trial of atezolizumab (1200 mg IV infusion on day 1 of each 21-day cycle) plus BCG (1 instillation/week) with an induction treatment period of 6 weeks, followed by a maintenance treatment period of 52 weeks maximum. During induction, dose-limiting toxicity (DLT) and maximum tolerated dose of atezolizumab were determined. The primary study outcome was recurrence-free survival (RFS).

**Results:**

A total of 36 patients with high-risk NMIBC with a median age of 70 years were enrolled in this study. Twenty participants completed the whole treatment schedule. No DLTs were recorded during the study period. The median RFS was not reached; RFS rates were 83% (95% CI 70.6%-95.4%) and 73% (95% CI 57.4%-88.2%) at the end of the first and second year, respectively. The incidence of serious drug-related adverse events was low. Additionally, no impairment in Health-related quality of life status throughout the study was observed.

**Conclusions:**

IV atezolizumab plus intravesical BCG in high-risk BCG-naïve NMIBC patients resulted in a high RFS rate and a manageable safety profile. These results appear consistent with those of the ALBAN study, which did not demonstrate a significant benefit of this combination over BCG alone.

Implications for Practice This is the second published trial evaluating the upfront combination of atezolizumab and BCG in high-risk BCG-naïve NMIBC. The results showed promising antitumor activity, with 2-year recurrence-free survival approaching 73% and no dose-limiting toxicities. The quality of life was slightly affected, and the safety profile was manageable. These findings support the rationale for combining immune checkpoint inhibitors with BCG in early-stage bladder cancer.

## Introduction

Atezolizumab is a fully humanized IgG1 monoclonal antibody targeting programmed death ligand 1 (PD-L1) currently approved in Europe but not in the US for locally advanced or metastatic urothelial bladder cancer (BC) based on the results of the IMvigor 210^1^^,^^2^ and IMvigor211 studies.^3^ Atezolizumab was further studied in patients with bacillus Calmette-Guérin (BCG)-unresponsive non-muscle invasive bladder cancer (NMIBC) in the phase II SWOG S1605 trial.^4^ In this trial, a complete response in carcinoma *in situ* (CIS) tumors was experienced by 41% and 27% of patients at 3 and 6 months, respectively. In addition, the 18-month event-free survival (EFS) rate was 29% in the overall population and 45% in the population with Ta/T1 disease. No unexpected toxicity was observed.^4^

Evidence from clinical trials combining BCG and PD-L1 inhibitors in NMIBC suggests synergistic potentiated antitumor activity.^5–7^ However, the potential synergistic effect of atezolizumab and BCG in BCG-naïve high-risk NMIBC patients had not yet been evaluated by the time our study was planned.^8^ BladderGATE was a phase Ib-II clinical trial designed to evaluate the safety, tolerability, patient-reported outcomes (PROs), and antitumor activity of upfront atezolizumab administered by intravenous (IV) infusion combined with intravesical BCG in patients with BCG-naïve high-risk NMIBC.

## Materials and methods

The BladderGATE trial was conducted at the Department of Urology of Hospital Universitario 12 de Octubre. The trial was sponsored by the ONCOSUR Foundation and registered at ClinicalTrials.gov (NCT04134000) and at the European Union Drug Regulating Authorities Clinical Trials Database (EudraCT No.: 2019-002061-36). The study was conducted in accordance with the Good Clinical Practice Guidelines and the ethical principles of the Declaration of Helsinki. All patients provided written informed consent before participating in the trial. The study protocol was reviewed and approved by the independent ethics committee of Hospital Universitario 12 de Octubre (Madrid, Spain; Code N. CEIm: 19/402).

### Study design and treatment

**Figure 1.**
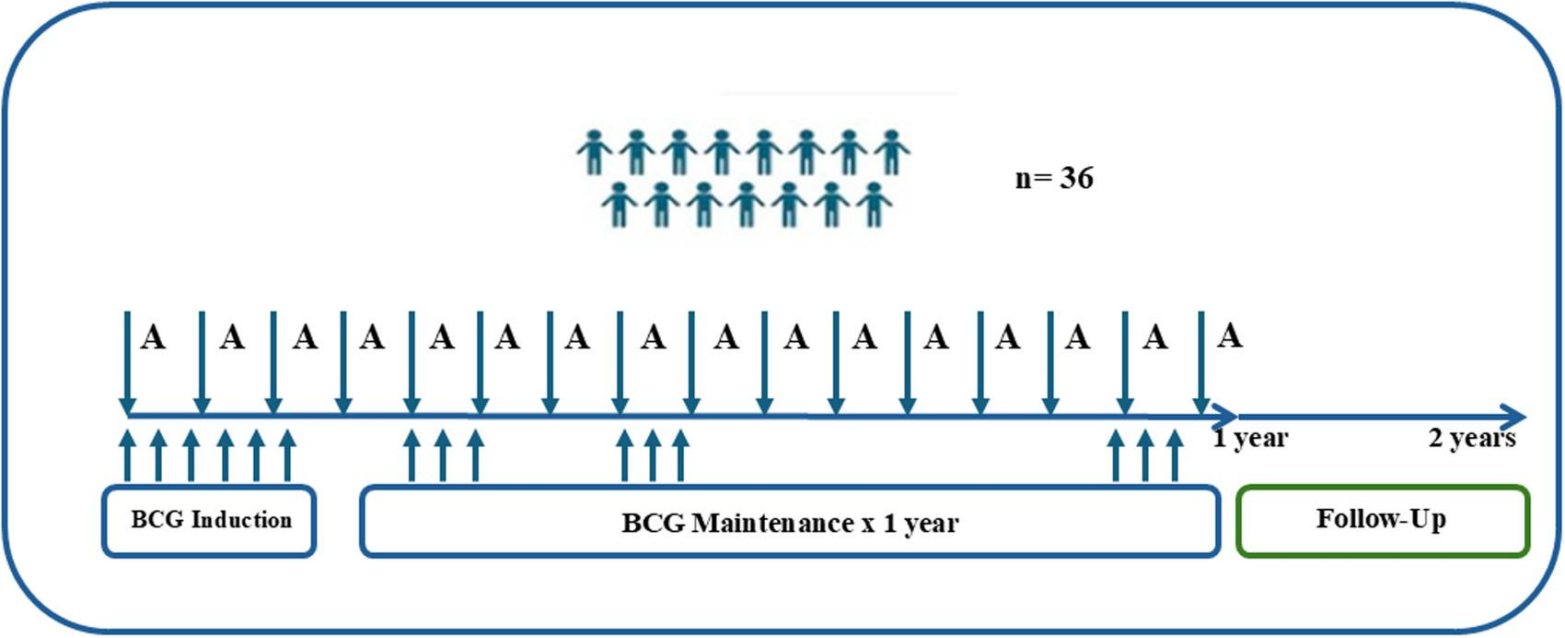
Study treatment schedule. Atezolizumab (A) 1200 mg IV day 1 for each 21-day cycle (maximum, 52 weeks). Bacillus Calmette–Guérin (BCG) was added weekly for 6 weeks plus weekly maintenance for 3 weeks at weeks 12, 24, and 48.

This was a phase Ib/II open-label, nonrandomized, dose de-escalation clinical trial of IV atezolizumab in combination with intravesical BCG in patients with high-risk BCG-naïve NMIBC. Atezolizumab 1200 mg was administered as an IV infusion on day 1 of each 21-day cycle, up to objective disease progression or unacceptable toxicity. The maximum duration of atezolizumab treatment was 52 weeks. Additionally, BCG was administered weekly during the induction treatment period of 6 weeks. Afterward, BCG was administered as a maintenance treatment once a week for 3 weeks at weeks 13, 14, and 15; at weeks 25, 26, and 27; and at weeks 49, 50, and 51 (Figure 1).

During the first de-escalation phase, the dose-limiting toxicity (DLT) and maximum tolerated dose (MTD) of atezolizumab were determined. Patients were assigned to each dose level in groups of 10 subjects until the MTD (the highest dose level at which less than 4 out of 10 patients experienced DLT) was achieved. The DLT was determined during the induction treatment period. The rules for de-escalation are presented in Table S1.

Once the MTD was reached, a further expansion phase was used to evaluate the safety and preliminary antitumor activity of the combination of atezolizumab and BCG. Between 10 and 20 patients were included in the de-escalation stage, whereas additional patients, up to the final sample of 36, were included in the expansion cohort.

### Selection criteria

Eligibility criteria were age ≥ 18 years old, confirmed histopathology of high-risk non-muscle invasive (ie, T1, high-grade Ta—G3- and/or CIS) transitional cell carcinoma of the bladder, according to EAU Guidelines on NMIBC.^9^ In addition, patients were BCG-naïve (they had never been treated with BCG or had stopped it more than 2 years ago), had an Eastern Cooperative Oncology Group performance status (ECOG PS) of 0-2, and had adequate hematologic and end-organ function. The full lists of the inclusion and exclusion criteria are listed in Tables S2 and S3, respectively.

### Study outcomes and assessments

There were 2 coprimary outcomes for the study: the primary study outcome in the de-escalation phase was to assess the incidence and nature of DLTs of the combination of BCG plus atezolizumab, as graded according to the fifth version of the National Cancer Institute Common Terminology Criteria for Adverse Events (NCI CTCAE v5.0).^10^ Similarly, the primary study outcome for the expansion phase was high-grade recurrence-free survival (RFS), defined as the time from the date of treatment initiation until the date of objective disease progression or recurrence, or death (due to any cause in the absence of progression), regardless of whether the patient withdrew from therapy or received another anticancer therapy prior to progression. At month 3, cystoscopy and urine cytology were performed according to EAU Guidelines.^9^ Follow-up was carried out with quarterly white-light cystoscopy and urine voiding cytology, a CT scan of the urinary tract at the screening and at the end of treatment, and biopsy or TURBT when clinically indicated. Random bladder biopsies were performed in cases of positive or suspicious urine cytology with no visible tumor during cystoscopy, according to the Paris System for Reporting Urinary Cytology.^11^

Secondary outcomes were: safety, according to the NCI-CTCAE v 5.0^10^; and patient-reported quality of life, as measured by the European Organization for Research and Treatment of Cancer Quality of Life Questionnaire-Core 30 (EORTC QLQ-C30)^12^; and the NMIBC-specific module (EORTC QLQ-NMIBC24).^13^

The study was complemented with exploratory outcomes aimed at characterizing the tumor microenvironment and its correlation with treatment response, not reported in this manuscript.

### Statistical analysis

Given the exploratory nature of the study, no formal sample size calculation was performed. Approximately 40 patients were expected to be enrolled in this study.

Descriptive statistics were used for all variables and are presented as dose-level groups. Continuous variables are summarized as the means, SD, medians, and IQRs. Categorical variables are displayed as absolute and relative frequencies.

Regarding the primary efficacy outcome, the median (95% CI) RFS was estimated using the Kaplan–Meier method; patients without documented progression or recurrence were censored at their last evaluation (either with CT/MRI or cystoscopy/urine cytology); patients were not censored at initiation of other anticancer therapies. Additionally, with respect to the EORTC QLQ-C30 and EORTC QLQ-NMIBC24 questionnaires, changes between the baseline and final scores were analyzed using the Student’s *t* test.

For the safety and tolerability assessments, the number and percentage of DLTs by grade and treatment were calculated. Adverse events were coded using the MedDRA dictionary V.27.0^14^ and they were presented with absolute and relative frequencies by dose level group according to the CTC-AE grade.^10^

The efficacy population consisted of all patients who met the selection criteria and received at least one dose of the study medication, whereas the safety population comprised all patients who received at least one dose of the study treatment.

Statistical analyses were performed using SPSS version 26 (IBM).

## Results

**Figure 2.**
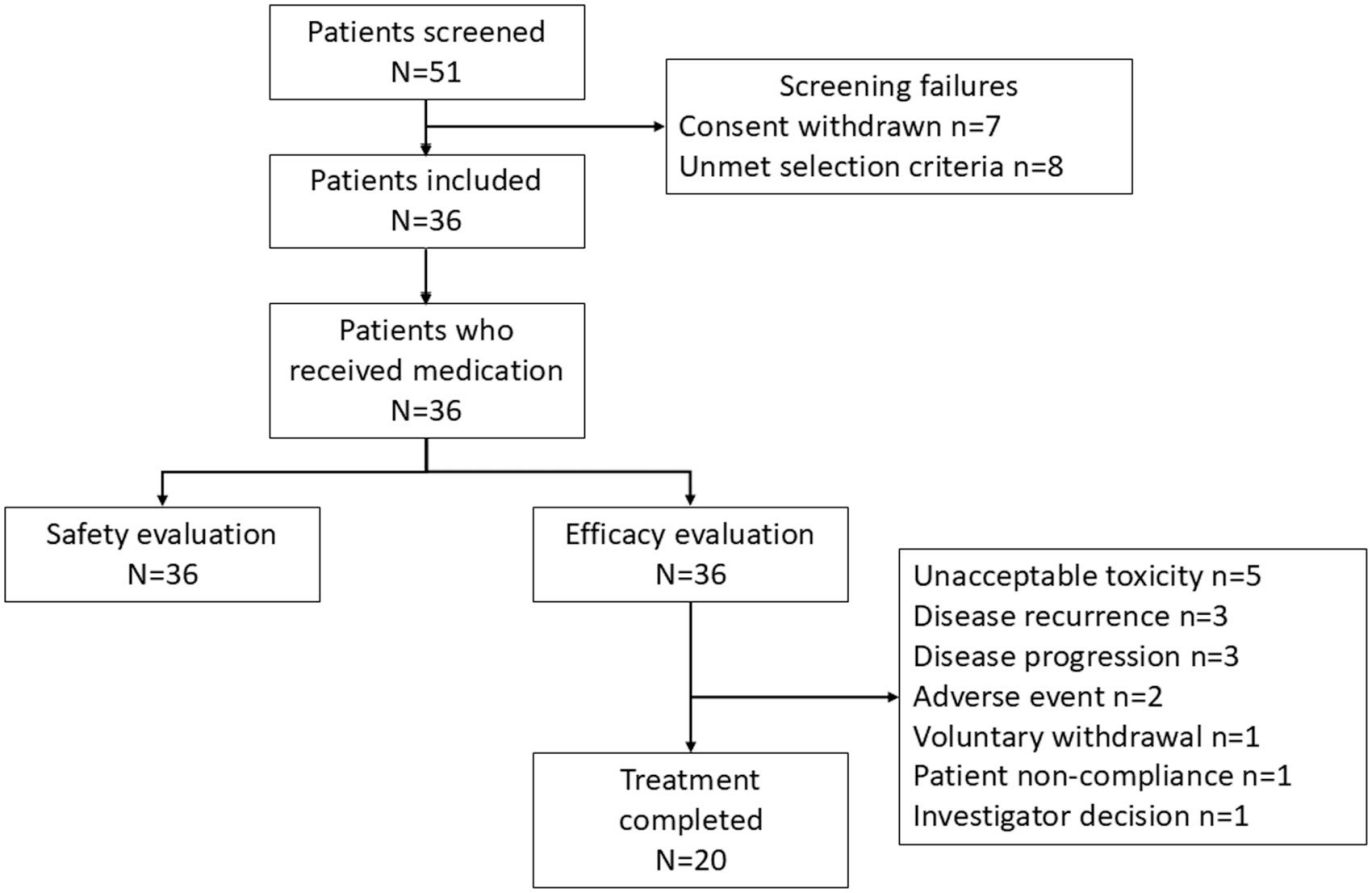
Patient distribution.

Between July 2020 and December 2022, 36 patients with high-risk NMIBC were enrolled in the study and received at least one dose of the atezolizumab and BCG combination. All 36 patients were included in the efficacy and safety analyses. Twenty of them completed the study treatment, while 16 discontinued it for several reasons, with the most frequent reasons being unacceptable toxicity (*N* = 5), disease recurrence, and disease progression (3 patients each) (Figure 2). Patients who discontinued atezolizumab treatment did not continue with BCG alone. Baseline characteristics are summarized in Table 1.

**Table 1.** Baseline demographic and clinicopathological characteristics.

Characteristics	*N* = 36
**Age, years, median (IQR)**	70.0 (63.5, 77.0)
**Sex, *n* (%)**	
** Male**	30 (83.3)
**Tumor stage, *n* (%)**	
** Ta**	16 (44.4)
** T1**	16 (44.4)
** CIS**	2 (5.6)
** Unknown^a^**	2 (5.6)
**Histology, *n* (%)**	
** Urothelial carcinoma**	24 (66.7)
** Histological subtype (<50% differentiation)**	12 (33.3)
**Differentiation grade, *n* (%)**	
** G1 (well differentiated)**	1 (2.8)
** G2 (moderately differentiated)**	8 (22.2)
** G3 (poorly differentiated)**	24 (66.7)
** Not available**	3 (8.3)
**ECOG PS, *n* (%)**	
** 0**	34 (94.5)
** 1**	2 (5.6)

Abbreviations: CIS, Carcinoma in situ; ECOG PS, Eastern Cooperative Oncology Group Performance Status; G, Tumor grade.

a Not available (1) and Tx (1).

The median (IQR) number of atezolizumab cycles was 15.5 (9.0, 17.0), the median (IQR) number of BCG doses was 12 (10.0, 15.0), and the median (IQR) exposure treatment time was 52.2 weeks (28.9, 54.1).

### Incidence of dose-limiting toxicity

No DLTs were recorded during the study period. Therefore, the initial dose of DL0 of BCG (1 instillation per week) combined with atezolizumab (1200 mg IV q3w) was maintained throughout the trial.

### Recurrence-free survival

**Figure 3.**
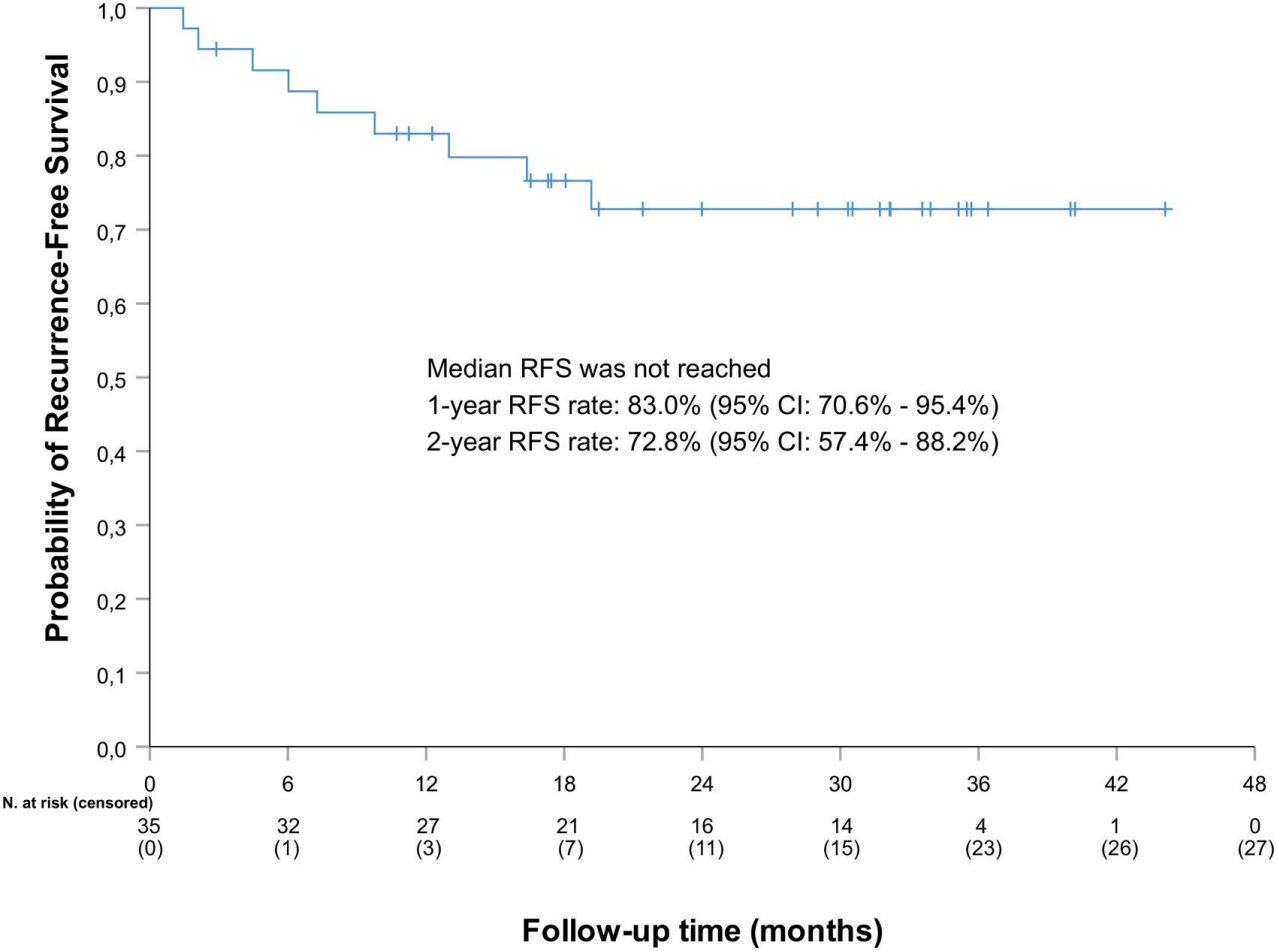
Recurrence-free survival. RFS, recurrence-free survival.

Over a median (IQR) follow-up time of 30.9 months (18.7, 34.8), the median RFS was not reached (Figure 3). The RFS rates were 83% (95% CI: 70.6%-95.4%) and 72.8% (95% CI: 57.4%-88.2%) at the end of the treatment period and at the end of follow-up (second year), respectively. The characteristics of patients presenting with either disease progression or recurrence are shown in Table S4.

### Health-related quality of life

The global health status score of the EORTC QLQ-C30 significantly decreased at the end of the study (−9.2 [22.0]; *P* < .05). There was a decrease in the scores for all domains of the Functional Scales, but the changes were not statistically significant (Table S5). We also observed an increase in the Symptom Scale scores, but they were statistically significant only for fatigue (9.6 [22.2]; *P* < .05), constipation (11.5 [29.9]; *P* < .05), and diarrhea (5.6 [12.6]; *P* < .05) (Table S5).

With respect to the EORTC QLQ-NMIBC24 questionnaire, the mean (SD) changes from baseline were statistically significant for the intravesical treatment issues (8.6 [22.7]; *P* < .05) and for the sexual enjoyment (−21.2 [27.0]; *P* < .05) domains (Table S6).

### Safety and tolerability

Adverse events (AEs) were reported by 35 patients (97.2%); most of them were grade 1 or 2, and none was grade 5. The most frequently recorded AEs were pruritus (30.6%), asthenia (27.8%), dysuria (27.8%), hypothyroidism (22.2%), pyrexia (22.2%), and urinary tract infections (22.2%) (Table 2). Two AEs of special interest were recorded: one conjunctival edema (grade 2) and one immune-mediated myocarditis (grade 3). Furthermore, 9 serious adverse events (SAEs), mostly grade 3, were reported by 9 patients. These patients required hospitalization, and all of them recovered from the AE. SAEs included lymphadenitis, acute myocardial infarction, adrenal insufficiency, influenza-like illness, chronic pyelonephritis, prostate cancer, immune-mediated encephalopathy, Guillain–Barré syndrome, and immune-mediated lung disease. Lymphadenitis and influenza-like illness were related to BCG, whereas adrenal insufficiency, immune-mediated lung disease, immune-mediated encephalopathy, and Guillain–Barré syndrome were considered related to atezolizumab (Table S7).

**Table 2.** Adverse events per patient (incidence ≥5%)^a^.

		Grade							
		1		2		≥ 3		Total	
SOC	PT	*N*	%	*N*	%	*N*	%	*N*	%
**Blood and lymphatic system disorders**	Lymphadenitis	1	2.8	0	0.0	1	2.8	2	5.6
	Leukocytosis	2	5.6	0	0.0	0	0.0	2	5.6
**Endocrine disorders**	Hypothyroidism^b^	4	11.1	4	11.1	0	0.0	8	22.2
	Hyperthyroidism	3	8.3	0	0.0	0	0.0	3	8.3
**Gastrointestinal disorders**	Dry mouth	3	8.3	1	2.8	0	0.0	4	11.1
	Rectal tenesmus	1	2.8	1	2.8	0	0.0	2	5.6
	Constipation	2	5.6	0	0.0	0	0.0	2	5.6
**General disorders and administration site conditions**	Asthenia	5	13.9	2	5.6	3	8.3	10	27.8
	Pyrexia	7	19.4	1	2.8	0	0.0	8	22.8
**Hepatobiliary disorders**	Hypertransaminasemia	2	5.6	3	8.3	0	0.0	5	13.9
	Hyperbilirubinemia	1	2.8	0	0.0	1	2.8	2	5.6
	Hepatitis	1	2.8	1	2.8	0	0.0	2	5.6
**Infections and infestations**	Urinary tract infection	0	0.0	8	22.2	0	0.0	8	22.2
	Cystitis	3	8.3	2	5.6	0	0.0	5	13.9
	Nasopharyngitis	2	5.6	0	0.0	0	0.0	2	5.6
**Investigations**	Aspartate aminotransferase increased	2	5.6	0	0.0	0	0.0	2	5.6
	Alanine aminotransferase increased	2	5.6	0	0.0	0	0.0	2	5.6
**Metabolism and nutrition disorders**	Hypophosphatasemia	2	5.6	0	0.0	0	0.0	2	5.6
	Hyponatremia	0	0.0	0	0.0	2	5.6	2	5.6
	Hyperglycemia	1	2.8	1	2.8	0	0.0	2	5.6
**Musculoskeletal and connective tissue disorders**	Myalgia	3	8.3	0	0.0	0	0.0	3	8.3
	Back pain	3	8.3	0	0.0	0	0.0	3	8.3
	Arthralgia	2	5.6	1	2.8	0	0.0	3	8.3
**Renal and urinary disorders**	Dysuria	7	19.4	3	8.3	0	0.0	10	27.8
	Pollakiuria	3	8.3	3	8.3	0	0.0	6	16.7
	Hematuria	4	11.1	1	2.8	0	0.0	5	13.9
	Polyuria	3	8.3	1	2.8	0	0.0	4	11.1
	Bladder irritation	2	5.6	1	2.8	0	0.0	3	8.3
	Urinary tract discomfort	2	5.6	0	0	0	0.0	2	5.6
	Nocturia	1	2.8	1	2.8	0	0.0	2	5.6
**Respiratory, thoracic and mediastinal disorders**	Cough	1	2.8	1	2.8	0	0.0	2	5.6
**Skin and subcutaneous tissue disorders**	Pruritus	10	27.8	1	2.8	0	0.0	11	30.6
	Rash	2	5.6	1	2.8	0	0.0	3	8.3
	Dermatitis	1	2.8	0	0.0	1	2.8	2	5.6
**Vascular disorders**	Hypertension	1	2.8	2	5.6	0	0.0	3	8.3

Abbreviations: PT, preferred term; SOC, system organ class.

a Percentages are calculated over the total number of valid patients for safety evaluation (*n* = 36).

b Only 1 patient temporarily discontinued treatment with atezolizumab. None of the 8 patients withdrew from the study.

Sixteen patients discontinued the study (Table S8). Five patients discontinued the study due to unacceptable toxicity and 2 patients owing to AEs, respectively (Table S9). One patient who withdrew from the study due to unacceptable toxicity died 2 years later as a result of disease progression. None of the patients discontinued the study due to COVID-19.

## Discussion

BladderGATE is a single-arm phase Ib/II trial that evaluated the efficacy and safety of atezolizumab combined with BCG in patients with high-risk BCG naïve NMIBC. The RFS rates after the first and second years were 83% (95% CI: 70.6%-95.4%) and 73% (95% CI: 57.4%-88.2%), respectively, and the median RFS was not achieved. In the recently published ALBAN study, which compared atezolizumab plus BCG versus BCG alone in patients with high-risk BCG-naïve NMIBC, the combination of atezolizumab and BCG did not show any benefit in its primary efficacy endpoint, EFS (HR, 0.98; 95% CI: 0.71-1.36), or in the secondary endpoint, RFS (HR, 1.06; 95% CI: 0.73-1.55).^8^ A visual estimation from its RFS survival curve suggests that the authors found RFS rates similar to the ones found in our study, and therefore our results appear consistent with those of the ALBAN study.^8^ Unlike the ALBAN study, the combination of other immune checkpoint inhibitors with BCG have demonstrated a higher efficacy versus BCG alone.^6^^,^^7^ A phase III randomized study revealed a statistically significant increase in EFS with the combination of sasanlimab, an anti-PD-1 monoclonal antibody, and BCG maintenance, compared with BCG alone (HR, 0.68; 95% CI: 0.49-0.94), in patients with high-risk BCG-naïve NMIBC (CREST study).^7^ Similarly, the results from another phase III trial, the POTOMAC study, which evaluated durvalumab, an anti-PD-L1 monoclonal antibody, combined with BCG, as induction with or without maintenance therapy in the same population, demonstrated a statistically significant improvement in disease-free survival compared with BCG induction and maintenance therapy alone (HR, 0.68; 95% CI: 0.50-0.93).^6^ Discrepancies between the ALBAN trial and the other 2 studies might be due to a drug-specific effect, although some other methodological aspects cannot be dismissed, such as trial design, endpoint definitions, and treatment duration. In this regard, the CREST and POTOMAC studies used 2 years of BCG maintenance,^6^^,^^7^ while in the ALBAN study there was only one year.^8^ Similarly, the duration of the treatment with checkpoint inhibition was 2 years for sasanlimab^7^ and 1 year for durvalumab and atezolizumab.^6^^,^^8^ A comparison of the characteristics of the aforementioned studies is presented in Table S10.

Our study presented a manageable safety profile. The DLT for BCG combined with atezolizumab (1200 mg IV q3w) was not reached; hence, the DLO was not de-escalated to DL1. Notably, no new safety alerts related to any of the study treatments were detected in the present study. Only 2 SAEs were related to BCG (lymphadenitis and influenza-like illness), and 4 were related to atezolizumab (adrenal insufficiency, immune-mediated lung disease, immune-mediated encephalopathy, and Guillain–Barré syndrome). These safety results are consistent with those reported in a previous trial evaluating atezolizumab plus BCG in patients with high-risk BCG-unresponsive NMIBC,^15^ and comparable with those described in the ALBAN study.^8^

In our study, health-related quality of life (HRQoL) was also assessed using 2 validated questionnaires: the EORTC QLQ-C30 and the EORTC QLQ-NMIBC24. Except for some slight impairments in specific domains, such as fatigue, constipation, intravesical treatment issues, and sexual enjoyment, there was no significant impairment in other HRQoL domains throughout the study. Interestingly, HRQoL, as measured by the EORTC QLQ-NMIBC24, was generally maintained throughout the entire CREST trial follow-up,^7^ a finding comparable to what we observed in BladderGATE.

This is the second published study assessing the combination of atezolizumab and BCG in high-risk BCG-naïve NMIBC patients, and its efficacy and safety results appear consistent with the findings of the ALBAN trial.^8^ However, it should bear in mind that the primary endpoint of the ALBAN study, EFS, was different to the one we used in the BladderGATE trial, RFS. Among the strengths of this study, it is worth mentioning the long follow-up duration of 2 years, considering that this was a phase Ib/II trial. Additionally, our study is one of the few published NMIBC trials that provide patient-reported outcomes prospectively, which gives this work a distinctive added value. However, this study has several limitations. This was an exploratory study with a small sample size. Furthermore, we must not dismiss a possible bias in the study results because of the high patient discontinuation rate (44.4%). Another limitation is the lack of a control group with BCG alone, which would better permit formal comparisons with the ALBAN study. In addition, the 1-year BCG maintenance duration might have been suboptimal, although it has been reported as a common approach.^16–18^ Finally, the negative impact of the COVID-19 pandemic on patient compliance in our study cannot be ignored. In fact, the patient recruitment for the study was hampered by the delays in disease diagnosis and the marked reduction in surgical activity caused by COVID-19.

The treatment landscape for BCG-naïve HR NMIBC patients is rapidly evolving,^5^^,^^17^ leading to some important challenges. Firstly, there is a need to find the right balance between efficacy and safety, improving survival,^19^ while reducing potential side effects.^20^ While the combination of systemic immune checkpoint inhibitors and intravesical BCG improves efficacy in BCG-naïve NMIBC patients, this benefit may be counterbalanced by a significantly higher risk of severe adverse events, a fact that emphasizes the need for a personalized, risk assessment and biomarker-driven approach to optimize treatment in these patients.^21^ Secondly, access to new therapies, limited by country-driven cost-containment policies, has become a major issue. Thirdly, there is an increasing demand for efficient collaboration between diverse specialists within multidisciplinary teams to optimize the care of these patients,^5^^,^^22^ as currently recommended by some consensus documents.^23^ Finally, there is a need for biomarkers that can define which patients will benefit from the above-mentioned combination therapy. In this sense, circulating tumor DNA (ctDNA) has been identified as a promising biomarker for predicting oncological outcomes in MIBC.^24^ Notably, in the recently published phase III trial, IMvigor011, patients with MIBC and positive ctDNA treated with adjuvant atezolizumab achieved significantly longer DFS and overall survival than those treated with placebo.^25^ Nonetheless, while ctDNA detection rates in patients with NMIBC are relatively low (approximately 52% of Ta), urinary tumor DNA (utDNA) has emerged as a more sensitive and highly effective biomarker for detecting minimal residual disease (MRD) and predicting recurrence in these patients.^26^

## Conclusions

Intravenous atezolizumab combined with intravesical BCG in high-risk BCG-naïve NMIBC patients resulted in a high RFS rate and a manageable incidence of drug-related adverse events. Our efficacy results appear consistent with those of the ALBAN study, which did not demonstrate a significant benefit of this combination over BCG alone. In the future, other combinations and/or studies with biomarker-driven patient selection should be conducted.

## Supplementary Material

oyag177_Supplementary_Data

## Data Availability

Data are available for bona fide researchers who request it from F.G.-R. and D.C.

## Clinical trial registration

This trial was registered at ClinicalTrials.gov (NCT04134000)

EudraCT No.: 2019-002061-36
