# Supplementary material for: The combination of atezolizumab and BCG in high-risk non-muscle invasive bladder cancer: results of the phase Ib/II BladderGATE clinical trial
Source: Oncologist. 2026 May 8;31(6):oyag177. doi: 10.1093/oncolo/oyag177 (PMC13218416; doi:10.1093/oncolo/oyag177)
Supplement: oyag177_Supplementary_Data [file oyag177_supplementary_data.doc]

**SUPPLEMENTARY INFORMATION**

**Supplementary Table 1.** Rules for dose de-escalation

| 1. If <4 patients experience DLT, de-escalation part will be terminated, as no escalation is planned abo 2. e DL-0. DL-0 h 3. s been the recommended do 4. e for expansion phase. |
| --- |
| 1. If 4 patients in the first cohort (DL-0) develop a DLT, further enrollment in that cohort will cease and ten patients will be included in the dose level below (DL-1), even if the 10 patients planned in the first cohort have not been recruited. |
| 1. If <4 patients experience DLT in DL-1, this dose level would be the recommended dose for expansion phase. |
| 1. If 4 of the first 10 evaluable patients in second cohort (DL-1) develop a DLT, further enrollment in that cohort could stop and the study terminated, as no de-escalation is planned below DL-1. |
| 1. The recommended dose for expansion phase will be the highest dose at which less than 4 of 10 evaluable patients in a given cohort experiences a DLT (DL-0). |
| 1. If <4 patients experience DLT, de-escalation part will be terminated, as no escalation is planned above DL-0. DL-0 has been the recommended dose for expansion phase. |
| 1. If 4 patients in the first cohort (DL-0) develop a DLT, further enrollment in that cohort will cease and ten patients will be included in the dose level below (DL-1), even if the 10 patients planned in the first cohort have not been recruited. |
| 1. If <4 patients experience DLT in DL-1, this dose level would be the recommended dose for expansion phase. |
| 1. If 4 of the first 10 evaluable patients in second cohort (DL-1) develop a DLT, further enrollment in that cohort could stop and the study terminated, as no de-escalation is planned below DL-1. |
| 1. The recommended dose for expansion phase will be the highest dose at which less than 4 of 10 evaluable patients in a given cohort experiences a DLT (DL-0). |

DLT, Dose-limiting toxicity

**Supplementary Table 2.** Study inclusion criteria

| 1. Patients ≥ 18 years old. |
| --- |
| 1. Signed Informed Consent Form |
| 1. Histologically confirmed diagnosis of high-risk non-muscle-invasive (T1, high grade Ta - G3- and/or carcinoma in situ) transitional cell carcinoma of the bladder |
| 1. Never treated with BCG or stopped >2 years ago |
| 1. ECOG PS 0-2 |
| 1. No prior radiation to bladder |
| 1. Life expectancy ≥ 5 years |
| 1. Adequate hematologic and end-organ function defined by the following laboratory results obtained within 14 days prior to the first study treatment: |
| a. ANC ≥ 1500/μL (without granulocyte colony-stimulating factor support within 2 weeks prior to the first dose of study treatment) |
| b. WBC counts > 2500/μL and < 15,000/μL |
| c. Lymphocyte count ≥ 300/μL |
| d. Platelet count ≥ 100,000/μL (without transfusion within 2 weeks prior to the first dose of study treatment) |
| e. Hemoglobin ≥ 9.0 g/dL. Patients may be transfused or receive erythropoietic treatment to meet this criterion |
| f. AST, ALT, and alkaline phosphatase ≤ 2.5 × ULN |
| g. Patients with known Gilbert disease who have serum bilirubin level ≤ 3 × ULN may be enrolled |
| h. INR and aPTT ≤ 1.5 × ULN. This applies only to patients who are not receiving therapeutic anticoagulation; patients receiving therapeutic anticoagulation should be on a stable dose. |
| i. Creatinine clearance ≥ 30 mL/min (calculated using the Cockcroft Gault formula |
| 1. The time elapsed between the TURBT and the start of the study treatment will not be less than 4 weeks or more than 12 weeks |
| 1. Women who are not postmenopausal (≥ 12 months of non−therapy-induced amenorrhea) or surgically sterile must have a negative serum pregnancy test result within 14 days prior to the first dose of study treatment |
| 1. For women of childbearing potential: agreement to remain abstinent or use contraceptive methods that result in a failure rate of < 1% per year during the treatment period and for at least 150 days after the last dose of study drug.   A woman is considered to be of childbearing potential if she is postmenarcheal, has not reached a postmenopausal state (≥ 12 continuous months of amenorrhea with no identified cause other than menopause), and has not undergone surgical sterilization (removal of ovaries and/or ut  rus).  Examples of contraceptive methods with a failure rate of < 1% per year include bilateral tubal ligation, male sterilization, establishing proper use of hormonal contraceptives that inhibit ovulation, hormone-releasing intrauterine devices, and copper intrauterine devices.  The reliability of sexual abstinence should be evaluated in relation to the duration of the clinical trial and the preferred and usual lifestyle of the patient. Periodic abstinence (e.g., calendar, ovulation, symptothermal, or postovulation methods) and withdr  wa  are not acceptable methods of contraception. |
| 1. Tumor tissue biopsy at study entry or availability of an archival specimen obtained within 2 months of study screening. |
| 1. Willingness to complete all study-related procedures including patient-reported questionnaires. |

aPTT, activated partial thromboplastin time; ALT, alanine aminotransferase; ANC, absolute neutrophil count; AST, aspartate aminotransferase; BCG, Bacillus Calmette–Guérin; ECOG PS, Eastern Cooperative Oncology Group performance status; INR, international normalized ratio; TURBT, transurethral removal of bladder tumor; ULN, upper limit of normal; WBC, white blood cell.

**Supplementary Table 3.** Study exclusion criteria

| 1. Muscle-invasive, locally advanced nonresectable, or metastatic urothelial carcinoma (i.e   T2, T3, T4, and/or stage IV). |
| --- |
| 1. Previous BCG within a 2-year period. |
| 1. Life expectancy < 5 years. |
| 1. ECOG PS 3 or 4. |
| 1. Known additional malignancy that is progressing or requires active treatment excepting basal cell carcinoma of the skin, squamous cell carcinoma of the skin that has undergone potentially curative therapy or in situ cervical cancer. |
| 1. Active autoimmune disease that has required systemic treatment in the past 2 years. |
| 1. Evidence of interstitial lung disease or active noninfectious pneumonitis. |
| 1. Active infection requiring systemic therapy in the last two weeks. |
| 1. Pregnant or breastfeeding or expecting to conceive within the projected duration of the trial through 150 days after the last dose of study treatment. |
| 1. Prior therapy with an anti-programmed cell death 1 (PD-1), anti-PD-ligand 2 (L2) agent, or with an agent directed to another coinhibitory T-cell receptor. |
| 1. Known human immunodeficiency virus (HIV). |
| 1. Known active Hepatitis B or C infection or tuberculosis. |
| 1. Received a live virus vaccine within 30 days of planned start of study treatment. |
| 1. Treatment with any approved anticancer therapy, including chemotherapy (systemic or intravesical), radiation therapy (to the bladder), or hormonal therapy within 3 weeks prior to the first dose of study treatment. |
| 1. Treatment with any other investigational agent or participation in another clinical trial with therapeutic intent within 4 weeks prior to the first dose of study treatment. |
| 1. Allergy or hypersensitivity to components of the atezolizumab or BCG formulation. |
| 1. Prior allogeneic stem cell or solid organ transplantation. |
| 1. History of idiopathic pulmonary fibrosis, organizing pneumonia (e.g., bronchiolitis obliterans), drug-induced pneumonitis, idiopathic pneumonitis, or evidence of active pneumonitis on screening chest CT scan. |
| 1. Serum albumin < 2.5 g/dL. |
| 1. Severe infections within 4 weeks prior to the first dose of study treatment |
| 1. Signs or symptoms of infection within 2 weeks prior to the first dose of study treatment. |
| 1. Treatment with therapeutic oral or intravenous antibiotics within 2 weeks prior to the first dose of study treatment. |
| 1. Significant cardiovascular disease, such as New York Heart Association cardiac disease (Class II or greater), myocardial infarction within the previous 3 months, unstable arrhythmias, or unstable angina. |
| 1. Major surgical procedure other than for diagnosis within 4 weeks prior to the first dose of study treatment, or anticipation of need for a major surgical procedure during the course of the study. |
| 1. Any other diseases, metabolic dysfunction, physical examination finding, or clinical laboratory finding giving reasonable suspicion of a disease or condition that contraindicates the use of an investigational drug or that may affect the interpretation of the results or render the patient at high risk from treatment complications. |
| 1. History of prior systemic BCG infection |

BCG, Bacillus Calmette–Guérin; CT, computed tomography; ECOG PS, Eastern Cooperative Oncology Group performance status.

**Supplementary Table 4. Characteristics of patients with disease recurrence or progression**

| **Subject ID** | **Sex** | **Age**  **(years)** | **TNM Stage^a^** | **Grade^b^** | **Event^c^** |
| --- | --- | --- | --- | --- | --- |
| 1 | Male | 68 | T1, Nx, Mx; stage: I | G3 | Disease progression |
| 2 | Female | 57 | T1, N0, M0; stage: I | G3 | Disease recurrence |
| 3 | Male | 77 | T1, Nx, Mx; stage: I | G3 | Disease recurrence |
| 4 | Male | 73 | T1, N0, M0; stage: I | G3 | Disease recurrence |
| 5 | Male | 69 | T1, N0, M0; stage: I | G3 | Disease recurrence |
| 6 | Male | 72 | Tis, N0, M0; stage: 0 | NA | Disease recurrence |
| 7 | Male | 88 | T1, N0, M0; stage: 0 | G3 | Disease recurrence |

^a^T (Tx, Ta, Tis, T1), N (Nx, N0), M (Mx, M0), Stage (0, 0a, I)

^b^G1 (Well differentiated), G2 (Moderately differentiated), G3 (Poorly differentiated), NA (Not available)

^c^Death; Progression disease; Recurrence disease

**Supplementary Table 5.** Changes in HRQoL assessed with the EORTC QLQ-C30 questionnaire^a^

| **Domain** | **N** | **Mean change^b^** | **SD** | **P value*** |
| --- | --- | --- | --- | --- |
| Global health status | 30 | -9.2 | 22.0 | **0.03** |
| **Functional scales** |  |  |  |  |
| Physical functioning | 30 | -4.9 | 14.2 | 0.07 |
| Emotional functioning | 29 | -2.3 | 17.4 | 0.48 |
| Role functioning | 30 | -7.8 | 23.5 | 0.08 |
| Cognitive functioning | 29 | -6.3 | 22.9 | 0.15 |
| Social functioning | 30 | -9.4 | 28.3 | 0.08 |
| **Symptoms scales** |  |  |  |  |
| Fatigue | 30 | 9.6 | 22.2 | **0.02** |
| Nausea and vomiting | 30 | 1.1 | 6.1 | 0.33 |
| Pain | 29 | 4.0 | 21.7 | 0.33 |
| Dyspnea | 30 | 4.4 | 19.0 | 0.21 |
| Insomnia | 30 | 3.3 | 33.2 | 0.59 |
| Appetite loss | 30 | 1.1 | 20.5 | 0.77 |
| Constipation | 29 | 11.5 | 29.9 | **0.04** |
| Diarrhea | 30 | 5.6 | 12.6 | **0.02** |
| Financial difficulties | 30 | 0 | 21.4 | >0.99 |

HRQoL, health-related quality of life; N, number of patients evaluated; n. s., not significant; SD, standard deviation.

^a^Changes from baseline at the end of treatment

^b^Positive mean changes denote worse HRQoL, except for global health status and functional scales

*Paired samples t test

**Supplementary Table 6.** Changes in HRQoL assessed with the EORTC QLQ NMIBC24 questionnaire^a^

| **Domain** | **N** | **Mean change^b^** | **SD** | **P value*** |
| --- | --- | --- | --- | --- |
| Urinary symptoms | 29 | -4.1 | 28.2 | 0.44 |
| Malaise | 31 | 4.3 | 14.9 | 0.12 |
| Future worries | 30 | -1.7 | 24.2 | 0.71 |
| Bloating and flatulence | 31 | 4.3 | 19.2 | 0.22 |
| Sexual function | 23 | -2.9 | 20.5 | 0.50 |
| Male sexual problems | 17 | -1.0 | 20.0 | 0.84 |
| Intravesical treatment issues | 31 | 8.6 | 22.7 | **0.04** |
| Sexual intimacy | 10 | 10.0 | 27.4 | 0.28 |
| Risk of contaminating a partner | 11 | 3.0 | 27.7 | 0.72 |
| Sexual enjoyment | 11 | -21.2 | 27.0 | **0.03** |
| Female sexual problems^c^ | ----- | ----- | ----- | ----- |

HRQoL, health-related quality of life; N, number of patients evaluated; n. s., not significant; SD, standard deviation.

^a^Changes from baseline at the end of treatment

^b^Positive mean changes denote worse HRQoL, except for sexual function and enjoyment.

^c^Since no baseline evaluation of female sexual problems was recorded, differences and contrasts cannot be provided.

*Paired samples t test

**Supplementary Table 7.** Serious adverse events

| **AE description** | **Grade** | **Relation with the medication** | **Seriousness criteria** | **Resolution** |
| --- | --- | --- | --- | --- |
| Lymphadenitis | 3 | BCG | Hospitalization | Recovered |
| Influenza-like illness | 3 | BCG | Hospitalization | Recovered |
| Adrenal insufficiency | 3 | Atezolizumab | Hospitalization | Recovered |
| Immune-mediated lung disease | 3 | Atezolizumab | Hospitalization | Recovered |
| Immune-mediated encephalopathy | 3 | Atezolizumab | Hospitalization | Recovered |
| Guillain‒Barre syndrome | 3 | Atezolizumab | Hospitalization | Recovered |
| Acute myocardial infarction | 3 | None | Hospitalization | Recovered |
| Prostate cancer | 3 | None | Hospitalization | Recovered |
| Chronic pyelonefritis | 2 | None | Hospitalization | Recovered |

AE, adverse event

**Supplementary Table 8. Study treatment discontinuations**

| **End of treatment reason** | **N** | **%** |
| --- | --- | --- |
| Complete treatment according to protocol | 20 | 55.6 |
| Unacceptable toxicity | 5 | 13.9 |
| Recurrence disease | 3 | 8.3 |
| Progression disease | 3 | 8.3 |
| Adverse event | 2 | 5.6 |
| Withdrawal from the study | 1 | 2.8 |
| Patient non-compliance | 1 | 2.8 |
| Investigator decision | 1 | 2.8 |

**Supplementary Table 9.** Treatment discontinuation due to adverse events (AEs) or unacceptable toxicity

| **End of treatment reason** | **Description** | **Grade** |
| --- | --- | --- |
| Unacceptable toxicity | Hyponatraemia | 4 |
| Unacceptable toxicity | Renal insufficiency | 3 |
| Unacceptable toxicity | Maculopustular erythematous lesions (buttock skin with psoriasis) | 3 |
| Unacceptable toxicity | Immune-mediated hepatitis | 3 |
| Unacceptable toxicity | Skin rash | 3 |
| AE | Emotional impairment related to neurological deterioration due to atezolizumab infusions, depression, and symptoms worsening | 1 |
| AE | Acute presumed immuno-mediated encephalopathy and hypophysitis | 4 |

AE, adverse event

**Supplementary Table 10. Comparison of the key characteristics of the** studies combining immune checkpoint inhibitors and BCG in BCG-naïve high-risk NMIBC

| **Study** | **BCG duration** | **ICI, duration** | **Primary endpoint** | **CIS (%)** |
| --- | --- | --- | --- | --- |
| BladderGATE | I, 6 weeks  M, 1 year | Atezolizumab, 1 year | RFS | 5.6 |
| ALBAN | I, 6 weeks  M, 1 year | Atezolizumab, 1 year | EFS | 39.1 |
| POTOMAC | I, 6 weeks  M, 2 years | Durvalumab, 1 year | DFS | 37 |
| CREST | I, 6 weeks  M, 2 year | Sasanlimab, 2 years | EFS | 14.8 |

BCG, Bacillus Calmette-Guérin; CIS, Carcinoma in situ; DFS, Disease-free survival; EFS, event-free survival; I, Induction; ICI, Immune checkpoint inhibitor; M, Maintenance; NMIBC, Non-muscle-invasive bladder cancer; RFS, Recurrence-free survival
